# Comparison of the 48-week efficacy between entecavir and adefovir in HBeAg-positive nucleos(t)ide-naïve Asian patients with chronic hepatitis B: a meta-analysis

**DOI:** 10.1186/1743-422X-8-75

**Published:** 2011-02-22

**Authors:** Pan Zhao, Weiwei Liu, Jun Zhao, Qun Guan

**Affiliations:** 1 Therapy and Research Center for Liver Failure, Beijing 302 Hospital, Beijing 100039, PR China; 2 Science and Technology Division, Academy of Military Medical Science, Beijing 100850, PR China

## Abstract

**Background:**

Although entacavir and adefovir were widely used in most Asian countries, there were few conclusions drawn from a meta-analysis for comparing the efficacy between entecavir and adefovir in nucleos(t)ide-naïve Asian patients with chronic hepatitis B (CHB). The aim of this study was to evaluate the 48-week efficacy between the two drugs in HBeAg-positive nucleos(t)ide-naïve Asian CHB patients with the method of Meta analysis, which was generally accepted by the international as the best evidence for evaluating the efficacy of drugs.

**Methods:**

We searched all data documented in Pubmed, Embase, Wanfang Database and CNKI (China National Knowledge Infrastructure) before November 30, 2010. Heterogeneity was examined by Chi-square test, the relative risk calculated and forest plot drawn. Rates of undetected serum HBV DNA, serum alanine aminotransferase (ALT) normalization, HBeAg clearance and HBeAg seroconversion were analyzed. A total of 6 articles was included. Meta analysis showed that the rate of undetected serum HBV DNA (relative risk, 1.73; 95% confidence interval, 1.38-2.17; *P *< 0.00001) and that of serum ALT normalization (relative risk, 1.25; 95% confidence interval, 1.06-1.49; *P *= 0.009) in the entecavir group were higher than those in the adefovir group. However, no statistic significance existed between the two groups in the rate of HBeAg clearance (relative risk, 0.77; 95% confidence interval, 0.44-1.35; *P *= 0.36), or the rate of HBeAg seroconversion (relative risk, 0.74; 95% confidence interval, 0.28-1.94; *P *= 0.53).

**Conclusions:**

Entecavir is superior to adefovir in decreasing serum HBV DNA and normalizing ALT but similar with adefovir in clearing HBeAg and encouraging HBeAg seroconversion for the HBeAg-positive nucleos(t)ide-naive Asian patients with chronic hepatitis B. Adefovir can be still used for first-line therapy in these patients.

## 1. Introduction

Infection with HBV is a major public health problem. Approximately 2 billion people have been exposed to HBV, and more than about 350 million are chronically infected with HBV [1]. Chronic hepatitis B (CHB) can lead to life-threatening conditions like liver cirrhosis (LC) and hepatocellular carcinoma (HCC) [2-4]. By the effective anti-HBV therapy, the worsening progress may be blocked or delayed. Oral nucleoside and nucleotide analogues (NAs) have revolutionized the treatment of chronic hepatitis B, which can suppress HBV replication in most patients and improve transaminase levels. To date, three nucleoside analogues (lamivudine, entecavir, telbivudine) and one nucleotide analogue (adefovir) are approved for the treatment of HBV infection in most of Asian countries. It can be confirmed that NAs have exhibited powerful strength in improving liver histology in most patients, however, two major shortcomings of NAs therapy frequently negate the benefits. One is the high rate of virological relapse when treatment is discontinued, and the other is the development of antiviral drug resistance when treatment is administered in long term [5]. Consequently, clinically relevant indicators for the efficacy in therapy of chronic hepatitis B are often a drop in circulating HBV DNA below detection level, clearance of HBeAg, seroconversion from HBeAg to corresponding anti-HBe antibodies, and normalization in serum ALT.

Recently, a Bayesian meta-analysis was performed in evaluating the efficacy among these approved NAs in nucleos(t)ide-naïve patients by Woo G [6]. The author limited the literature search to the English language, and did not classify the patients according to HBV endemic regions. However, it was known to us, Asian CHB patients possessed self-characteristics different from western populations. For example, the durability of hepatitis B e antigen responses after a period of therapy is lower in Asian populations than that in western populations [7]. So, it was necessary to re-evaluate the efficacy of NAs in Asian populations. Although entacavir and adefovir were widely used in most Asian countries, there are few conclusions drawn from a meta-analysis for comparing the efficacy between entecavir and adefovir in nucleos(t)ide-naïve Asian CHB patients. The aim of study was to evaluate the 48-week efficacy between the two drugs in HBeAg-positive nucleos(t)ide-naïve Asian CHB patients with the method of Meta analysis, which was generally accepted by the international as the best evidence for evaluating the efficacy of drugs.

## 2. Data and methods

### 2.1 Literature Search

We searched Pubmed, Embase, Wanfang Database and CNKI (National Knowledge Infrastructure) from the date of inception until November 30, 2010. Of these databases, Wanfang Database and CNKI provided literatures in Chinese. In this study, the search was designed using "entecavir", "adefovir", "chronic hepatitis B", "lamivudine resistant or lamivudine refractory". Reference lists from retrieved documents were also searched.

### 2.2. Inclusion and exclusion criteria

All the following criteria were included: (i) study design: randomized controlled trial; (ii) study population: HBeAg-positive nucleos(t)ide-naïve Asian CHB patients with HBeAg positivity; (iii) intervention: the doses of entecavir and adefovir were respectively 0.5 mg/d and 10 mg/d, with the duration lasting 48 weeks.

The exclusion criteria were as follows: (i) non-human studies; (ii) coinfection with hepatitis A, C, D, E, Epstein-Barr virus, cytomegalovirus or HIV; (iii) coexistence of any other liver diseases such as autoimmune hepatitis, alcoholic liver disease, drug hepatitis or Wilson's disease; (IV) liver transplantation; (V) past or current hepatocellular carcinomas. Literatures with only abstracts provided were also excluded.

### 2.3. Data extraction

Data were independently extracted from each study using pre-defined forms by two investigators (Pan Z and Weiwei L), and disagreement was resolved by discussion among investigators and reference to the original article. When several publications pertaining to a single study were identified, the most complete publication was used. The following information was extracted: the study design (including random sequence generation, blind method, and description of withdrawals and dropouts); patient characteristics; number of cases and controls; the concrete study results.

### 2.4. Efficacy measures and definitions

All outcome measurements were intermediate end points taken at 48 weeks, because it is appreciated that some patients would be continued or discontinued on oral therapy beyond this time period. Data extracted included rates of virological and biochemical response, HBeAg clearance, and HBeAg seroconversion. Virological response was defined as attainment of undetectable levels of serum HBV DNA. Biochemical response was defined as normalization of serum ALT. HBeAg clearance was defined as HBeAg disappearance and HBeAg seroconversion was defined as anti-HBe appearance.

### 2.5. Data analysis

Data analysis was carried out with the use of Review Manager Software 4.2 (Cochrane Collaboration, Oxford, United Kingdom). For each eligible study, dichotomous data were presented as relative risk (RR), and both with 95% confidence intervals (CI). Meta-analysis was performed using fixed-effect or random-effect methods, depending on the absence or presence of significant heterogeneity. Statistical heterogeneity between trials was evaluated by the chi-square and I-square (I^2^) tests. In the absence of statistically significant heterogeneity, the fixed-effect method was used to combine the results. When heterogeneity was confirmed (*P *< 0.05), the random-effect method was used. The overall effect was tested using *Z *scores, with significance set at *P *< 0.05.

## 3. Results

### 3.1. Characteristic and Quality of Studies

Of the 658 studies we identified in the search, 398 and 260 articles were published in English and Chinese, respectively. After a review of the full texts, 652 articles were excluded and 6 articles [8-13] (1 in English and 5 in Chinese) were included based on the pre-specified criteria. One of the 6 articles written by Leung N et al, was a international multi-center study and inevitably included non-Asian patients. However, through inquisition into the detailed information from related persons working in Bristol-Myers Squibb, we confirmed that only minor non-Asian patients (12%) were included in the study designed by Leung N et al [10], and the number of these patients in ETV group was equal to that in ADV group. So, we included this high-quality study after discussion. The characteristics of the 6 clinical trials included were shown in Table 1.

**Table 1:** Characteristic of the included studies

literature	patient races	study design
Ding H, 2005 [8]	Asian	randomized controlled study with description of withdrawals and dropouts
Zhang Q, 2009 [9]	Asian	randomized controlled study with description of withdrawals and dropouts
	88% Asian	
Leung N, 2009 [10]	12% non-Asian	randomized controlled study with description of withdrawals and dropouts
Yang F, 2010 [11]	Asian	randomized controlled study with description of withdrawals and dropouts
Zou S, 2010 [12]	Asian	randomized controlled study with description of withdrawals and dropouts
Huang H, 2010 [13]	Asian	randomized controlled study with description of withdrawals and dropouts

### 3.2. Virological response

**Figure 1:**
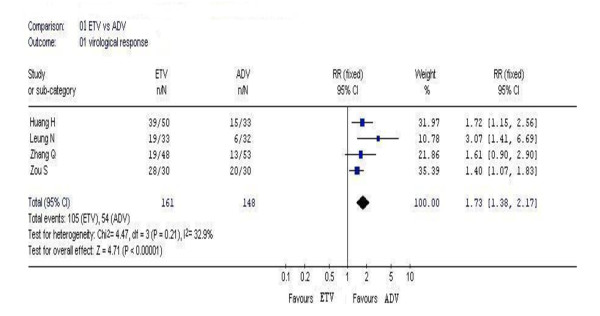
Forest plot-analysis of the 48-week virological response of entecavir therapy versus adefovir therapy

In this analysis, 4 studies reported the rates of undetected serum HBV DNA. According to chi-squared statistic and I square, heterogeneity was assessed and not found to be a concern. Greater virological response rates were observed in the entecavir group as compared with that in the adefovir group, and the difference in the rate between two groups were statistically significant [105/161 vs. 54/148, *RR *= 1.73, 95%CI (1.38-2.17), *P *< 0.00001](Figure 1).

### 3.3. Biochemical response

**Figure 2:**
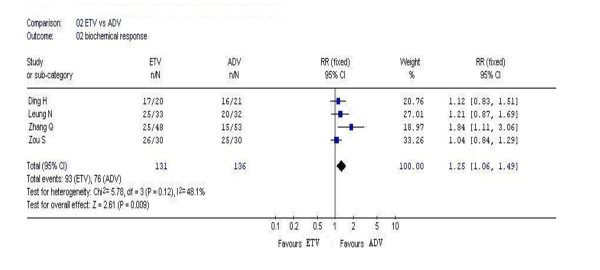
Forest plot-analysis of the 48-week biochemical response of entecavir therapy versus adefovir therapy

In this analysis, 4 studies reported the rates of serum ALT normalization. According to chi-squared statistic and I square, heterogeneity was assessed and not found to be a concern. The biochemical response rates in the entecavir group was higher as compared with that in the adefovir group, and the difference in the rate between two groups were statistically significant [93/131 vs. 76/136, *RR *= 1.25, 95%CI (1.06-1.49), *P *= 0.009](Figure 2).

### 3.4. HBeAg clearance

**Figure 3:**
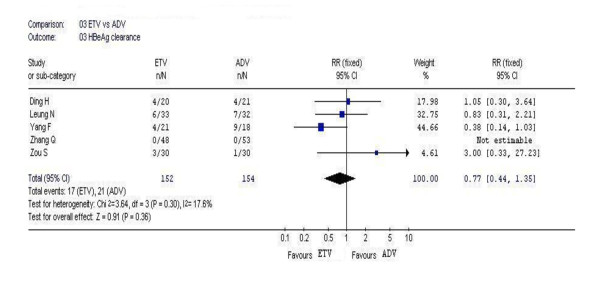
Forest plot-analysis of the 48-week HBeAg clearance of entecavir therapy versus adefovir therapy

In this analysis, 5 studies reported the rates of HBeAg clearance. According to chi-squared statistic and I square, heterogeneity was assessed and not found to be a concern. However, the difference in the rates of HBeAg clearance at week 48 between the two groups became similar, and no statistic significances existed [17/152 vs. 21/154, *RR *= 0.77, 95%CI (0.44-1.35), *P *= 0.36](Figure 3).

### 3.5. HBeAg seroconversion

**Figure 4:**
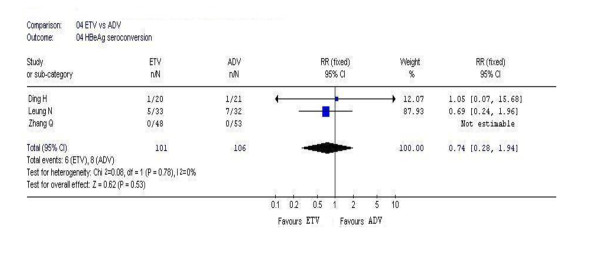
Forest plot-analysis of the 48-week HBeAg seroconversion of entecavir therapy versus adefovir therapy

In this analysis, 3 studies reported the rates of HBeAg seroconversion. According to chi-squared statistic and I square, heterogeneity was assessed and not found to be a concern. However, the difference in the rates of HBeAg seroconversion at week 48 between the two groups were also similar, and no statistic significances existed [6/101 vs. 8/106, *RR *= 0.74, 95%CI (0.28-1.94), *P *= 0.53](Figure 4).

## 4. Discussion

Adefovir is an acyclic monophosphate adenine analogue which was approved for the treatment of chronic hepatitis B at a dose of 10 mg/day in 2002. Worldwide, there have been an estimated 410,000 patient-years of adefovir use through 2008 [14]. ADV is a potent and widely-used anti-HBV drugs in Asian countries, and this drug has potent antiviral efficacy in nucleoside-naïve patients with CHB, resulting in significant virological, biochemical, and histological improvement [15-18]. A randomized, double-blind, placebo-controlled, prospective study conducted by Dong PL et al, showed that ADV could effectively suppress HBV DNA and normalize ALT at week 48 [19]. Entecavir is a carboxylic 2^'^-deoxyguanosine analogue, and is approved in the US, EU and many Asian countries [20]. The recommended once-daily oral dosage of entecavir is 0.5 mg in nucleos(t)ide-naïve patients. In the ETV-023 study conducted in China, entecavir treatment provided better efficacy than lamivudine at 48 weeks in terms of the composite primary endpoint in a mixed population of HBeAg-positive or -negative patients [21].

In our study, we compared the efficacy between the two drugs in suppressing HBV DNA, normalizing ALT, clearing HBeAg and encouraging HBeAg seroconversion with the method of meta-analysis, and found out that for the HBeAg-positive nucleos(t)ide-naive Asian CHB patients, ETV exhibited better efficacy than ADV at 48 weeks in suppressing HBV DNA and normalizing ALT, however, in clearing HBeAg and encouraging HBeAg seroconversion, the efficacy between the two drugs was similar. It is a consensus that clearance of HBeAg and/or development of anti-HBe are associated with improved outcomes [22], so, for Asian population, ADV treatment is not inferior to ETV treatment in all aspects for assessment of anti-HBV therapy. Owing to the cost benefit in the long-term experience and safety, particularly in Asian countries [23], it is confirmed that ADV can be still used for first-line therapy in the HBeAg-positive nucleos(t)ide-naive patients with CHB.

In conclusion, entecavir is superior to adefovir in decreasing serum HBV DNA and normalizing ALT, but similar with adefovir in clearing HBeAg and encouraging HBeAg seroconversion for the HBeAg-positive nucleos(t)ide-naive Asian patients with chronic hepatitis B. Adefovir can be still used for first-line therapy in these patients.

## Competing interests

The authors declare that they have no competing interests.

## Authors' contributions

PZ and JZ performed the majority of analyses; WWL provided analytical tools and was involved in the analysis; PZ designed the study and wrote the manuscript; JZ and QG were involved in editing the manuscript. All authors read and approved the final manuscript.
